# Assessment for Melting Temperature Measurement of Nucleic Acid by HRM

**DOI:** 10.1155/2016/5318935

**Published:** 2016-10-19

**Authors:** Jing Wang, Xiaoming Pan, Xingguo Liang

**Affiliations:** College of Food Science and Engineering, Ocean University of China, Qingdao 266003, China

## Abstract

High resolution melting (HRM), with a high sensitivity to distinguish the nucleic acid species with small variations, has been widely applied in the mutation scanning, methylation analysis, and genotyping. For the aim of extending HRM for the evaluation of thermal stability of nucleic acid secondary structures on sequence dependence, we investigated effects of the dye of EvaGreen, metal ions, and impurities (such as dNTPs) on melting temperature (*T*_*m*_) measurement by HRM. The accuracy of HRM was assessed as compared with UV melting method, and little difference between the two methods was found when the DNA *T*_*m*_ was higher than 40°C. Both insufficiency and excessiveness of EvaGreen were found to give rise to a little bit higher *T*_*m*_, showing that the proportion of dye should be considered for precise *T*_*m*_ measurement of nucleic acids. Finally, HRM method was also successfully used to measure *T*_*m*_s of DNA triplex, hairpin, and RNA duplex. In conclusion, HRM can be applied in the evaluation of thermal stability of nucleic acid (DNA or RNA) or secondary structural elements (even when dNTPs are present).

## 1. Introduction

High resolution melting (HRM) is a robust technique for mutation scanning, methylation, and genotyping, which possesses high sensitivity to distinguish DNA species from tiny differences in their melting temperatures based on sequence composition, length, GC content, or strand complementarity [1–4]. To obtain precise *T*_*m*_, data are required to be collected at narrow temperature increments of 0.02~0.2°C. Due to the high sensitivity to small variations, HRM can distinguish small differences in nucleic acid sequences, even for single nucleotide polymorphism (SNP) [5, 6]. Meanwhile, rapid measurement and analysis become easy due to the high-throughput work platforms (96 or 384 wells) coupled with HRM-compatible software. Therefore, HRM can be speculated as a potential approach for fast, accurate, and high-throughput evaluation of the thermal stability of nucleic acid, in the case that the conventional UV melting method does not work [7].

However, at present, HRM has been mainly applied to distinguish two DNA species longer than 60 bp in PCRs. It is unknown whether it can be applied to the high-throughput evaluation of thermal stability of short DNA or RNA sequences, which provide significant information for investigating sequence dependence or predication of secondary structures. From the viewpoint of basic data for thermodynamics, there has been no specific report on measuring *T*_*m*_ of short DNA sequences by HRM, not to mention RNA [8]. In this study, we checked whether HRM could measure *T*_*m*_s of short DNA duplex, triplex, or hairpin and even RNA duplex. We found that *T*_*m*_s of DNA duplexes with various GC contents and lengths (even shorter than 10 bp) could be well measured by HRM. Effects of the dye and metal ion as well as other composites (such as deoxyribonucleoside triphosphates and free single strands) were also investigated.

## 2. Materials and Methods

### 2.1. Materials and Reagents

DNA and RNA oligonucleotides (in Tables 1 and 2) were synthesized from Invitrogen (Shanghai, China); the fluorescent dye EvaGreen 20x was obtained from Biotium (Hayward, CA).

### 2.2. *T*_*m*_ Measurement Using HRM Method

DNA duplex, DNA hairpin, or RNA duplex was prepared in a solution containing 10 mM phosphate buffer (pH 7.4), EvaGreen (0.25x, 0.5x, 1x, 2x, or 5x), and NaCl (0-1 M). For experiments involving Mg^2+^, the buffer of 10 mM Tris-HCl (pH 7.1), 1x EvaGreen, and Mg^2+^ (1–100 mM) was used. For DNA triplex, DNA oligomers were prepared in a solution containing 1x EvaGreen, 2.5 mM KCl, 10 mM Na_2_HPO_4_, 2 mM KH_2_PO_4_, 10 mM MgCl_2_, and 0.5 mM spermine (pH 5.5).

For fluorescence melting profiles, the mixed oligomer solution (10 *μ*L) was pipetted into 96-well microtiter plates; then, the microtiter plates were transferred to a PikoReal Real-Time PCR instrument (Thermo Scientific, Finland). Annealing was performed with a cooling rate of 0.1°C/s from 95°C to 10°C; then, fluorescence data were collected over a temperature range of 10–95°C in 0.1°C increments (the holding time was 2 seconds). All fluorescence data were acquired from at least three parallel tests in one plate and repeated twice.

### 2.3. *T*_*m*_ Measurement Using UV Melting Method

UV melting assays were operated by a UV-Vis Spectrophotometer (UV-1800, SHIMADZU, Tokyo, Japan). For *T*_*m*_ measurement of DNA duplexes, each DNA duplex (1 *μ*M) was dissolved in a buffer as the cases of HRM, but with no EvaGreen. The solution was added into a cuvette with 1 mm or 10 mm path lengths. Melting profiles were obtained at 260 nm over the temperature ranges from 95°C to 10°C and then from 10°C to 95°C at a ramp rate of 1°C/min. All UV melting assays were performed for three times at least, and *T*_*m*_s were calculated by the first derivatives of melting curves.

## 3. Results

### 3.1. Evaluation of *T*_*m*_ Measurement by HRM

In this study, EvaGreen was adopted as the fluorescent dye in *T*_*m*_ measurement by HRM, which was one of the most popular saturating dyes for HRM analysis. The detailed effects of DNA sequences, metal ions, and other factors on *T*_*m*_ measurement by HRM were investigated.

#### 3.1.1. The Limit of DNA Duplex Length for HRM

**Figure 1:**
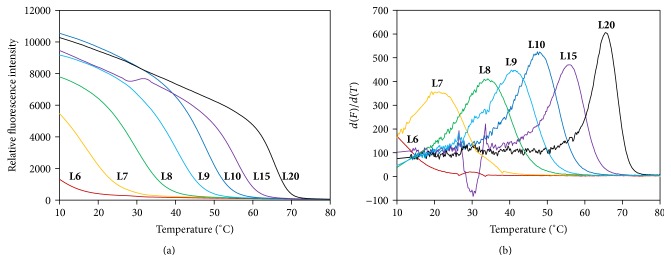
Fluorescence melting curves (a) and differential curves (b) of 6–20 bp DNA duplexes. Each DNA duplex (1 *μ*M) was measured in a 10 *μ*L solution containing 1x EvaGreen, 10 mM phosphate (pH 7.4), and 100 mM NaCl.

In order to investigate effects of DNA duplex length on *T*_*m*_ measurement by HRM, we used a series of DNA duplexes with lengths from 6 bp to 20 bp (**L6**–**L10**,** L15**, and** L20** in Table 1). The melting profiles of DNA duplexes are shown in Figure 1(a). The initial relative fluorescence intensity of 8–20 bp duplexes was at the range of 8000–11000, the intensity for the 7 bp duplex declined to about 5500, and the intensity of the 6 bp one was only about 1300, indicating that the 6 bp duplex was hard to be stained. The differential curves shown in Figure 1(b) also confirmed that only the melting peak of the 6 bp duplex was not detected and calculated. Therefore, EvaGreen can be used to measure *T*_*m*_ of 7 bp or longer DNA duplex by HRM.

#### 3.1.2. Effect of EvaGreen Concentration on *T*_*m*_ Measurement by HRM

**Figure 2:**
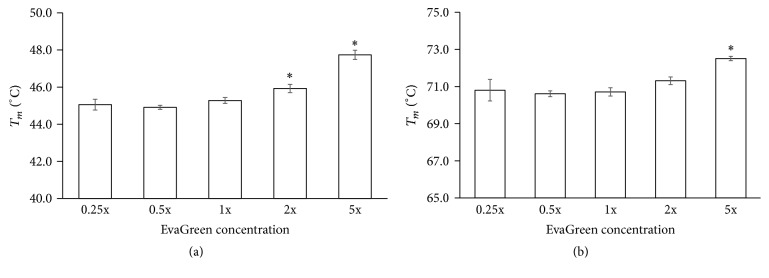
Effect of EvaGreen concentration on *T*_*m*_ of DNA duplex by HRM. DNA duplexes** GC3/15** (a) or** GC12/15** (b) of 1 *μ*M were measured in the solution with 0.25x, 0.5x, 1x, 2x, or 5x EvaGreen, 10 mM phosphate (pH 7.4), and 100 mM NaCl. Comparisons were done using one-way ANOVA analysis; ^*∗*^ *P* < 0.05 versus “0.05x” (*n* = 6).

The fluorescent dye concentration is an important factor for HRM analysis [9, 10]. When the dye is unsaturated, the dissociated dye from melted duplexes may reincorporate into other DNA duplexes, resulting in less fluorescent signal change and obtaining a higher *T*_*m*_. In order to evaluate the effect of EvaGreen concentration on *T*_*m*_ measurement by HRM, two sequences of** GC3/15** (20% GC content) and** GC12/15** (80% GC content) were used (Table 1). The obtained *T*_*m*_s for various EvaGreen concentrations (0.25x, 0.5x, 1x, 2x, or 5x) were shown in Figure 2. Interestingly,** GC3/15** (1 *μ*M) showed a lowest *T*_*m*_ when the EvaGreen was 0.5x (Figure 2(a)). For higher concentrations, 2x or 5x, *T*_*m*_ became higher. In the case of 5x, *T*_*m*_ was even 2-3°C higher than that of 0.5x. At 0.25x concentration, *T*_*m*_ became a little bit higher. This indicated that 0.5x EvaGreen was sufficient to bind with 1 *μ*M 15 bp DNA duplex. Similar results were also obtained for duplex** GC12/15** (Figure 2(b)).

Meanwhile, the DNA concentration effect on HRM *T*_*m*_ was also investigated. We measured the DNA duplexes** GC3/15** and** GC12/15** at various strand concentrations from 0.1 to 10 *μ*M in 1x EvaGreen by HRM. *T*_*m*_s by HRM were compared with that predicted by* mfold*, which is a popular software for calculating *T*_*m*_ (see Supplementary Figure S1 in Supplementary Material available online at http://dx.doi.org/10.1155/2016/5318935). When the DNA concentration was less than 1 *μ*M, the *T*_*m*_ difference between two methods was less than 2.5°C. The *T*_*m*_ difference became greater as DNA concentration increased to 5 or 10 *μ*M. Obviously, for a higher concentration of DNA, more EvaGreen is required, and a reasonable proportion of dye to DNA is essential for *T*_*m*_ measurement by HRM.

#### 3.1.3. Evaluation of *T*_*m*_ Accuracy by HRM

**Figure 3:**
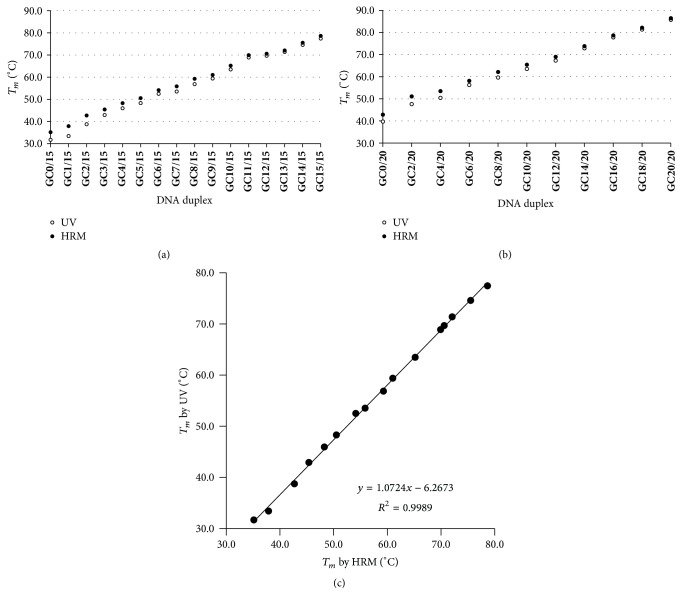
Comparison of *T*_*m*_ values of DNA duplexes measured by HRM and UV melting method. *T*_*m*_s of 15 bp (a) or 20 bp (b) DNA duplexes were measured, and the linear relationship between *T*_*m*_s of 15 bp DNA duplexes by HRM and UV was obtained (c).

To apply HRM to measure *T*_*m*_ of nucleic acid, its accuracy is a necessary aspect to be investigated by comparison with the traditional UV melting method. Two series of 15 bp or 20 bp DNA duplexes with various GC contents (*T*_*m*_ range of 30–90°C, Supplementary Tables S1 and S2) were measured by HRM and UV melting method, respectively. Some single stranded DNA may form secondary structures (e.g., the dG of F or R strand homodimer of **GC14/15** were lower than −22 kcal/mol); however, we found that they did not affect the *T*_*m*_ measurement of the duplex by HRM (Supplementary Figure S2). As shown in Figure 3(a) (15 bp DNA duplexes) and Figure 3(b) (20 bp DNA duplexes), there was little *T*_*m*_ difference between the two methods when *T*_*m*_ was higher than 40°C, although the difference became greater at the lower *T*_*m*_ range. In detail, for DNA duplexes with *T*_*m*_s higher than 70°C, their *T*_*m*_s by HRM were almost consistent with those by UV melting method. For *T*_*m*_s of 40–65°C, the *T*_*m*_ differences were 1-2°C. For *T*_*m*_s lower than 40°C, the *T*_*m*_ difference was within 4°C. We also found that there was a good linear relationship between *T*_*m*_s by the two methods (Figure 3(c)) which was used as the standard curve to correct HRM *T*_*m*_ data. Then, this linear formula was adopted to correct the HRM *T*_*m*_s of 20-bp DNA duplexes, and the results showed that the error of most corrected data was within 0.9°C as compared with UV data (Supplementary Table S2) and that the root mean-squared deviation (RMSD) of corrected *T*_*m*_ was 0.5°C, less than that of* mfold T*_*m*_ to UV *T*_*m*_ (1.7°C).

#### 3.1.4. Effect of Na^+^ or Mg^2+^ on *T*_*m*_ Measurement by HRM

**Figure 4:**
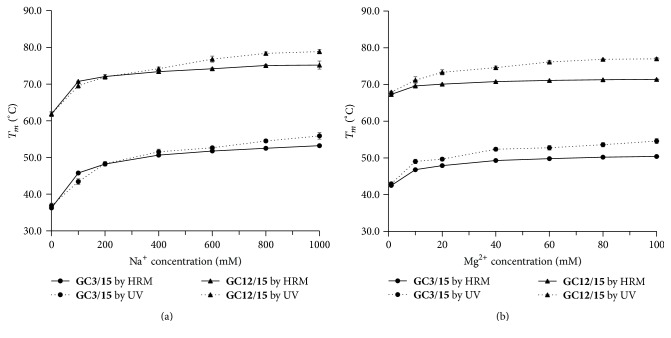
Effect of Na^+^ or Mg^2+^ on *T*_*m*_ measurement by HRM. *T*_*m*_s of DNA duplex** GC3/15** and** GC12/15** were measured by HRM with NaCl at various concentrations from 0 to 1 M (a) or with MgCl_2_ from 1 to 100 mM (b). The corresponding *T*_*m*_s were measured by UV.

In terms of the metal ion effect, we measured DNA duplexes** GC3/15** and** GC12/15** at various concentrations of Na^+^ or Mg^2+^ by HRM (Figure 4). HRM *T*_*m*_s of both** GC3/15** and** GC12/15** increased greatly with Na^+^ concentration from 0 to 100 mM, and the increasement became slightly at the range from 100 to 1000 mM (Figure 4(a)). The same trend was also observed for *T*_*m*_s obtained by UV, reflecting the effect of Na^+^ on stabilization of DNA duplex. When the Na^+^ was lower than 400 mM, almost the same *T*_*m*_ values were obtained by UV and HRM. When the Na^+^ was higher than 400 mM, *T*_*m*_s by UV became higher than those by HRM, and the difference increased with ion strength. According to their fluorescence melting curves (data not shown), the initial fluorescence intensity was lower when Na^+^ was at 0.6–1.0 M. The lower *T*_*m*_ by HRM indicated that Na^+^ at a high concentration affected the binding of EvaGreen to DNA.

The effect of Mg^2+^ concentration was shown in Figure 4(b). Here, the Tris-HCl buffer was used instead of phosphate buffer because magnesium phosphate has a low solubility. As compared with NaCl (Figure 4(a)), the difference between HRM and UV became much greater, especially at higher concentrations of MgCl_2_. Even at 10 mM MgCl_2_, *T*_*m*_ of** GC3/15** by HRM was 2.7°C lower than that by UV. Similar results were also obtained for** GC12/15**, and the difference between two methods became even greater. These results indicated that Mg^2+^ competed for DNA binding with EvaGreen containing only one positive charge; then, EvaGreen dissociated at a lower temperature.

#### 3.1.5. Effect of dNTPs on HRM

**Figure 5:**
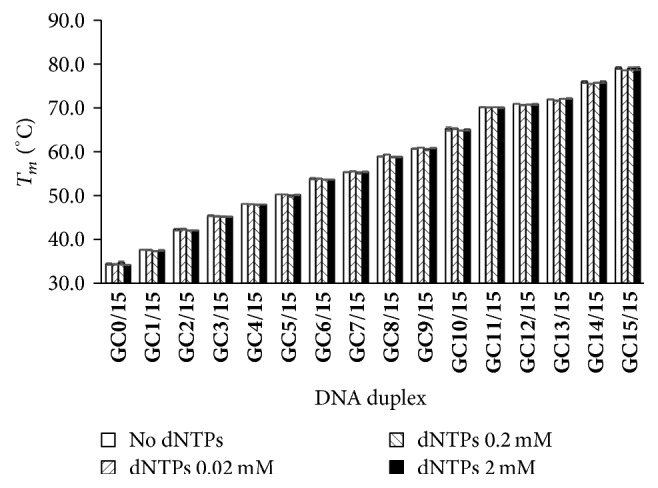
Effect of dNTPs on *T*_*m*_ measurement of DNA duplexes by HRM. The buffer contains 1x EvaGreen, 10 mM phosphate buffer (pH 7.4), and 100 mM NaCl. In this assay, 0, 0.02, 0.2, or 2 mM dNTPs were used.

In some biological reactions, dNTPs were present in the solution. Because dNTPs have UV absorption at 260 nm, *T*_*m*_ measurement of DNA duplexes by UV melting method becomes difficult. Whether dNTPs can affect *T*_*m*_ measurement by HRM was determined. In the presence of 0.02–2.0 mM dNTPs, *T*_*m*_s of** GC0/15**~**GC15/15** with various GC contents were measured, and almost no *T*_*m*_ change was observed at various concentration of dNTPs (Figure 5). However, high concentration of dNTPs (≥0.2 mM) resulted in excessively high absorbance (Supplementary Figure S3), beyond the accurate scale of UV spectrophotometers.

### 3.2. HRM Applied in DNA Triplex, Hairpin, and RNA Duplex

**Figure 6:**
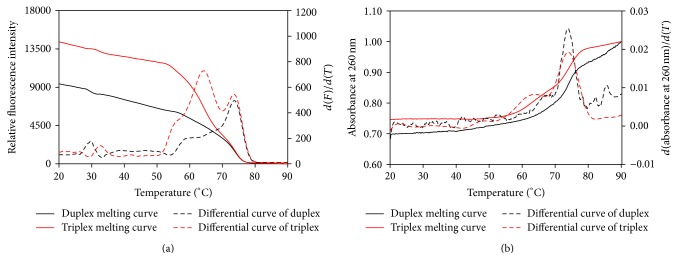
*T*_*m*_ measurement of DNA triplex by HRM (a) and by UV melting method (b). DNA duplex (black) and triplex (red) were detected in the same buffer solution (1 *μ*M of each strand, 2.5 mM KCl, 10 mM Na_2_HPO_4_, 2 mM KH_2_PO_4_, 10 mM MgCl_2_, and 0.5 mM spermine (pH 5.5)).

Except for DNA duplex, *T*_*m*_ measurement of DNA triplex and hairpin was also investigated by HRM. We constituted a DNA triplex (**Tri-a/a**′**/b** in Table 2) and identified it by UV melting method and HRM. As shown in Figure 6(a), two melting regions occurred in the fluorescence melting curve, which indicated that the *T*_*m*_s of duplex and triplex were 73.9 and 64.5°C. This was in line with the UV melting curves (Figure 6(b)) indicating that *T*_*m*_s of duplex and triplex were 73.7 and 63.9°C, respectively. Thus, HRM could detect the formation of DNA triplex.

In terms of DNA hairpin, we investigated a series of triloop hairpins possessing the same loop sequence, GAA, but differing in their length of stems (Supplementary Figure S4). We found that when the stem was 5 bp or longer, *T*_*m*_s could be obtained and the full width at half maximum of peak heights was as narrow as 10–12°C. It can be concluded that HRM can measure the *T*_*m*_ of a DNA hairpin as short as 5 bp. In order to further investigate whether HRM can detect the effect of the loop sequence on the *T*_*m*_ of a hairpin, we measured the GAA-loop hairpin (**HP-S6**, Table 2) and its mutants** HP-M1**~**M4** with loops of “IAA” “GIA” “GAI” or “IAI” (where I, inosine, served to replace the certain base of GAA) by HRM. The obtained *T*_*m*_s of IAA-, GAI-, and IAI-loop hairpins (65.2 ± 0.5, 69.7 ± 0.2, and 65.7 ± 0.4°C, resp.) were much lower than those of GAA- and GIA-loop hairpins (72.9 ± 0.3, 72.5 ± 0.2°C). This was in line with Moody and Bevilacqua's findings by UV melting method indicating that the first and third base of loop dominated the stability of DNA triloop hairpin [11, 12]. Thereby, this method can exactly evaluate the stability of short DNA hairpin on sequence dependence.

Considering the significance of RNA SSEs, we measured the *T*_*m*_ of a 21-bp RNA duplex (**RR**, Table 1) by HRM. It was found that** RR** presented good fluorescence melting curves at 1 *μ*M and 10 *μ*M, and the full width at half maximum of peak heights was only 5–5.5°C (Supplementary Figure S5). It was interesting that EvaGreen could bind to A-form conformation of RNA duplex. Therefore, HRM could be applied in *T*_*m*_ analysis of short RNA duplex, or other RNA secondary structures.

## 4. Discussion

In this study, whether HRM could measure *T*_*m*_s of small nucleic secondary structures was investigated. Surprisingly, HRM can measure as short as 7-bp DNA duplex with *T*_*m*_ of about 20°C (Figure 1). For the first time, HRM was proved to be capable of measuring the *T*_*m*_ of DNA triplex and short DNA hairpin (Figure 6). For hairpin structures, a minihairpin with 5 bp stem and 3 nt loop could also be analyzed by HRM. It can be concluded that HRM is suitable for evaluation of thermal stability of most secondary structural elements. Moreover, HRM measurement performs better stability and reproducibility compared with UV melting method. The standard deviation of the average *T*_*m*_ by HRM was within 0.5°C, lower than that by UV melting method (within 1.0°C) (Supplementary Table S1). Because the work platform of 96- or 384-well plate could be used for HRM and the data could be analyzed by well-edited processing software, HRM has been used as a high-throughput approach for determining SNP, gene methylation, and mutant analysis on a large scale [13–15]. Obviously, based on our study, HRM shows the potential for high-throughput evaluation of nucleic acid secondary structural elements, which is significant to the thermodynamic studies of nucleic acid. *T*_*m*_s of thousands of DNA sequences can be obtained in hours, even using 96-well plates (Supplementary Figure S6).

Another advantage of *T*_*m*_ measurement by HRM is that it is less affected by the presence of dNTPs (Figure 5). In routine PCRs or other approaches, the concentration of dNTPs is normally more than 0.2 mM [16–19]. However, UV melting method is hard to be used if the solution has compositions with strong UV absorption at 260 nm, such as enzymes and other additives in the buffer [20, 21]. Taken together, it can be concluded that HRM is suitable for measurement of practical *T*_*m*_ values in most biological reactions.

In terms of *T*_*m*_ measurement by HRM, some points should be noted including the proportion of dye to nucleic acid, the metal ion concentration, and other compositions. In some cases, the *T*_*m*_s obtained by HRM were higher than that of UV melting method, especially for low *T*_*m*_s (Figures 3(a) and 3(b)). The difference may be explained from two aspects. On the one hand, the binding of dye with positive charge can enhance the stability of DNA duplex, resulting in the fact that the dissociation of EvaGreen from the DNA duplex may occur at a relatively higher temperature. On the other hand, EvaGreen can bind to ssDNA, especially at a temperature below 40°C, and EvaGreen may remain binding with dissociated DNA, resulting in the fluorescence change delay and higher *T*_*m*_. Fortunately, there was a good linear relationship (the slope = 1.07, *R* ^2^ = 0.9989) between HRM *T*_*m*_ and UV *T*_*m*_ (Figure 3(c)), which can be used as the standard curve to correct HRM *T*_*m*_s (Supplementary Table S2). Therefore, HRM can be utilized as an accurate approach to evaluate the thermal stability of nucleic acids.

Another important point to be considered is the proportion of dye to nucleic acid when highly accurate *T*_*m*_ values are required [8, 9]. Our results showed that both the insufficiency and excessiveness of dye gave rise to a higher *T*_*m*_ (Figure 2 and Supplementary Figure S1). In addition, the metal ion at high concentrations may affect the accuracy of HRM. When the Na^+^ concentration was higher than 400 mM or Mg^2+^ higher than 10 mM, HRM *T*_*m*_s remained almost constant (Figure 4), which did not conform to the fact that nucleic acid with high concentration of metal ions shows higher *T*_*m*_ [22, 23]. This may be attributed to the competition between Na^+^ (or Mg^2+^) and EvaGreen for binding with DNA. Accordingly, HRM using EvaGreen is not suitable for the studies of salt dependence of nucleic acid stability [24, 25]. However, in most biological reactions, the Na^+^ concentration is lower than 400 mM, and Mg^2+^ concentration is not higher than 10 mM; thus, the accuracy of HRM is acceptable for most cases.

## 5. Conclusions

In conclusion, HRM can be used as a high-throughput approach for *T*_*m*_ measurement of small nucleic acid secondary structures, including minihairpins, triplex, or RNA structures. The high throughput of this approach makes it possible for obtaining large amounts of data, for example, the sequence dependence of nucleic acid secondary structure. In fact, the work of thermodynamic evaluation of DNA and RNA hairpins by HRM is being carried out by our group and some valuable characteristics of sequence dependence have been obtained. It can be expected that HRM will play an important role in the thermodynamic study of nucleic acid.

## Supplementary Material

The supplementary material contains additional data used for assessing the melting temperature measurement of nucleic acid by HRM. Supplementary Tables S1 and S2 supply the detailed melting temperature (*T*_*m*_) data of 15 bp and 20 bp DNA duplexes, respectively. The *T*_*m*_ difference between UV and HRM in Table S1 indicated that HRM measurement performed better stability and reproducibility compared with UV melting method. Moreover, the linear formula obtained from Table S1 can be used to correct the HRM *T*_*m*_s of 20-bp DNA duplexes (Table S2), and the error of most corrected data was within 0.9^o^C as compared with UV data. The HRM *T*_*m*_ of DNA duplex **GC3/15** and **GC12/15** at various strand concentrations from 0.1 to 10 μM in 1× EvaGreen was investigated (Figure S1).The *T*_*m*_ difference between HRM and prediction became greater as DNA concentration increased to 5 or 10 μM. The results demonstrated that a reasonable proportion of dye to DNA is essential for *T*_*m*_ measurement by HRM. The HRM melting curves of **GC14/15** duplex, **GC14/15** duplex with additional F or R strand, and only F strand or R strand were investigated and the results showed that the single strands did not affect the *T*_*m*_ measurement of the duplex by HRM (Figure S2). For the UV melting method, it was found that dNTPs (≥0.2 mM) resulted in a high absorbance beyond the accurate scale of UV spectrophotometers (Figure S3). Melting curves of a series of triloop hairpins with 3~8-bp stem and a 21-bp RNA duplex were detected by HRM (Figures S4 and S5), which demonstrated that HRM could be applied in *T*_*m*_ analysis of DNA and RNA secondary structural elements. For the melting curves of 96 samples on a 96-well plate by HRM (Figure S6), all the data could be obtained in 1 to 2 h. The results showed its high efficiency and high throughput.

## Figures and Tables

**Table 1:** Sequences of DNA and RNA duplexes used in this study.

Name	Sequence of duplex (5′-F strand-3′/3′-R strand-5′)
**L6**	CTATCC/GATAGG
**L7**	CTATCAC/GATAGTG
**L8**	CGTATCAC/GCATAGTG
**L9**	CGTCATCAC/GCAGTAGTG
**L10**	CGTCATCAGC/GCAGTAGTCG
**L15**	GCCCTGGTGATTAAA/CGGGACCACTAATTT
**L20**	GGGTGCCGTATTGACAAAAC/CCCACGGCATAACTGTTTTG
**GC0/15**	TAAAATAATAATAAT/ATTTTATTATTATTA
**GC1/15**	TATAAATAAGTAAAT/ATATTTATTCATTTA
**GC2/15**	AAGTACATTTATATA/TTCATGTAAATATAT
**GC3/15**	AATAAACATTCTCAA/TTATTTGTAAGAGTT
**GC4/15**	GTTTTTCAGTGAATA/CAAAAAGTCACTTAT
**GC5/15**	GGTTTTTCAGTGAAT/CCAAAAAGTCACTTA
**GC6/15**	AGAGGTTTTTCAGTG/TCTCCAAAAAGTCAC
**GC7/15**	GCCCTGGTGATTAAA/CGGGACCACTAATTT
**GC8/15**	CTCACGCCTGTAATC/GAGTGCGGACATTAG
**GC9/15**	GAGTCTCGCTCTGTC/CTCAGAGCGAGACAG
**GC10/15**	TGGCACCGAGGTGAC/ACCGTGGCTCCACTG
**GC11/15**	TGCGTGGCACCGAGG/ACGCACCGTGGCTCC
**GC12/15**	GAGGTGCACCGCCGC/CTCCACGTGGCGGCG
**GC13/15**	CGGCGCCCTCGCTCC/GCCGCGGGAGCGAGG
**GC14/15**	GCCGCGGCGCCCTCG/CGGCGCCGCGGGAGC
**GC15/15**	GGCCGGCCGCGGCGC/CCGGCCGGCGCCGCG
**GC0/20**	AATAATAATAATAATAATAT/TTATTATTATTATTATTATA
**GC2/20**	ATTTTCTATTTTTTTAACTT/TAAAAGATAAAAAAATTGAA
**GC4/20**	AAAAACAGAAGTAAGATAAT/TTTTTGTCTTCATTCTATTA
**GC6/20**	CCGTATTGACAAAACATTAA/GGCATAACTGTTTTGTAATT
**GC8/20**	TGTCCTTCCGAGTATGATAT/ACAGGAAGGCTCATACTATA
**GC10/20**	GGGTGCCGTATTGACAAAAC/CCCACGGCATAACTGTTTTG
**GC12/20**	GAGGGAGCAGGAAGATCCGT/CTCCCTCGTCCTTCTAGGCA
**GC14/20**	GGAAGATCCGTGCGGCACCG/CCTTCTAGGCACGCCGTGGC
**GC16/20**	GCGCCCTCGCTCCTCGCCCT/CGCGGGAGCGAGGAGCGGGA
**GC18/20**	GGCCGCGGCGCCCTCGCTCC/CCGGCGCCGCGGGAGCGAGG
**GC20/20**	CGGGGCCGGCCGCGGCGCCC/GCCCCGGCCGGCGCCGCGGG
**RR**	CUGACCUAUGAAUUGACAGCC/GACUGGAUACUUAACUGUCGG

**Table 2:** Sequences of DNA triplex and hairpins used in this study.

Name	Sequence (5′-3′)
**Tri-a**	CATTGCGGAGAAAGAGAAAGAAAAACCTCCCT
**Tri-a**′	AGGGAGGTTTTTCTTTCTCTTTCTCCGCAATG
**Tri-b**	CTCTTTCTCTTTCTTTTTCT
**Hp-S6**	ctatccGAAggatag
**Hp-M1**	ctatccIAAggatag
**Hp-M2**	ctatccGIAggatag
**Hp-M3**	ctatccGAIggatag
**Hp-M4**	ctatccIAIggatag
