# Supplementary material for: Assessment for Melting Temperature Measurement of Nucleic Acid by HRM
Source: J Anal Methods Chem. 2016 Oct 19;2016:5318935. doi: 10.1155/2016/5318935 (PMC5090098; doi:10.1155/2016/5318935)
Supplement: Supplementary file 1 — The supplementary material contains additional data used for assessing the melting temperature measurement of nucleic acid by HRM. Supplementary Tables S1 and S2 supply the detailed melting temperature (T m) data of 15 bp and 20 bp DNA duplexes, respectively. The T m difference between UV and HRM in Table S1 indicated that HRM measurement performed better stability and reproducibility compared with UV melting method. Moreover, the linear formula obtained from Table S1 can be used to correct the HRM T ms of 20-bp DNA duplexes (Table S2), and the error of most corrected data was within 0.9oC as compared with UV data. The HRM T m of DNA duplex GC3/15 and GC12/15 at various strand concentrations from 0.1 to 10 μM in 1× EvaGreen was investigated (Figure S1).The T m difference between HRM and prediction became greater as DNA concentration increased to 5 or 10 μM. The results demonstrated that a reasonable proportion of dye to DNA is essential for T m measurement by HRM. The HRM melting curves of GC14/15 duplex, GC14/15 duplex with additional F or R strand, and only F strand or R strand were investigated and the results showed that the single strands did not affect the T m measurement of the duplex by HRM (Figure S2). For the UV melting method, it was found that dNTPs (≥0.2 mM) resulted in a high absorbance beyond the accurate scale of UV spectrophotometers (Figure S3). Melting curves of a series of triloop hairpins with 3~8-bp stem and a 21-bp RNA duplex were detected by HRM (Figures S4 and S5), which demonstrated that HRM could be applied in T m analysis of DNA and RNA secondary structural elements. For the melting curves of 96 samples on a 96-well plate by HRM (Figure S6), all the data could be obtained in 1 to 2 h. The results showed its high efficiency and high throughput. [file 5318935.f1.pdf]

Supplementary Information

Assessment for melting temperature measurement of nucleic acid by HRM

Jing Wang, Xiaoming Pan, Xingguo Liang*

College of Food Science and Engineering, Ocean University of China, Qingdao, 266003, China

* Corresponding author. *Email address:* liangxg@ouc.edu.cn

Supplementary Table S1 Comparison of reproducibility between HRM and UV melting method

	HRM			UV melting method		
	Av. T_m (^oC)	S.D. (^oC)	CV (%)	Av. T_m (^oC)	S.D. (^oC)	CV (%)
**GC0/15**	34.6	0.5	1.6	31.7	0.6	1.9
**GC1/15**	37.9	0.5	1.2	33.4	0.2	0.6
**GC2/15**	42.7	0.3	0.8	38.8	0.2	0.6
**GC3/15**	45.4	0.2	0.5	42.9	0.7	1.7
**GC4/15**	48.3	0.3	0.6	46.0	0.1	0.3
**GC5/15**	50.5	0.3	0.6	48.3	0.2	0.4
**GC6/15**	54.1	0.2	0.4	52.5	0.6	1.1
**GC7/15**	55.9	0.3	0.5	53.5	0.3	0.6
**GC8/15**	59.3	0.3	0.4	56.9	0.5	1.0
**GC9/15**	61.0	0.3	0.5	59.4	0.6	1.0
**GC10/15**	65.2	0.2	0.3	63.5	0.4	0.7
**GC11/15**	69.9	0.1	0.2	68.9	0.2	0.2
**GC12/15**	70.6	0.3	0.5	69.7	1.0	1.4
**GC13/15**	72.1	0.2	0.3	71.4	1.0	1.5
**GC14/15**	75.5	0.2	0.3	74.6	0.5	0.6
**GC15/15**	78.7	0.4	0.5	77.4	0.2	0.3

Av. T_m, S.D. and CV denote the average T_m, standard deviation of the average T_m and coefficient of variation, respectively.

Supplementary Table S2 Comparison of UV T_m and HRM T_m of 20 bp DNA duplex

DNA duplex	UV T_m [a] (°C)	mfold T_m [b] (°C)	HRM T_m [c] (°C)	T_m^{mod} [d] (°C)	ΔT_m [e] (°C)
**GC0/20**	39.7	43.5	42.8	39.7	-0.1
**GC2/20**	47.6	48.4	51.1	48.5	-0.9
**GC4/20**	50.4	53.3	53.5	51.1	0.6
**GC6/20**	56.3	56	58.1	56.1	-0.2
**GC8/20**	59.6	60.4	62.1	60.4	0.7
**GC10/20**	63.5	62.7	65.4	63.9	0.4
**GC12/20**	67.3	67.3	69	67.7	0.4
**GC14/20**	72.8	71.3	73.8	72.9	0.1
**GC16/20**	77.8	75.7	78.7	78.1	0.3
**GC18/20**	81.4	80.9	82.2	81.8	0.5
**GC20/20**	85.7	84.4	86.5	86.5	0.7
RMS[f]		1.7	2.1	0.5	

[a] UV T_m denotes T_m measured by UV melting method; [b] mfold T_m denotes T_m calculated in mfold web server; [c] HRM T_m denotes T_m measured by HRM; [d] T_m^{mod} denotes the corrected T_m, T_m^{mod} = 1.0724 * HRM T_m – 6.2673; [e] $\Delta T_m = T_m^{mod}$ - UV T_m; [f] RMSD denotes the root mean-squared deviation of HRM T_m or T_m^{mod} to UV T_m.

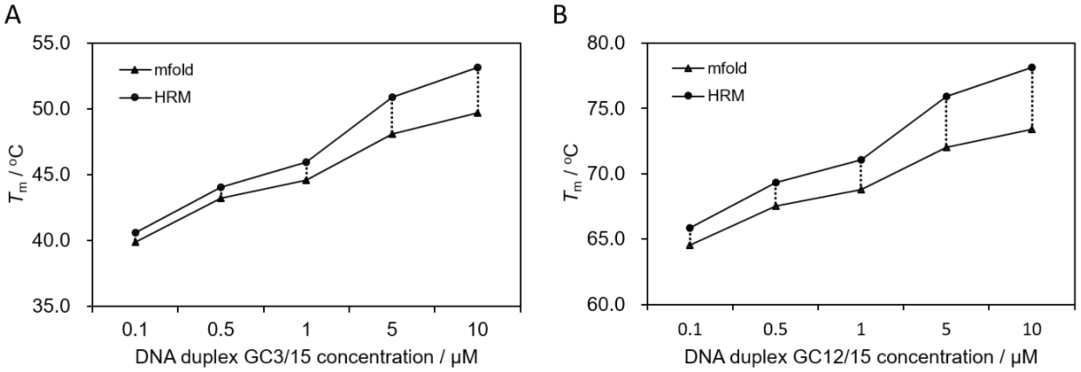

Supplementary Figure S1. T_ms of DNA duplexes at various strand concentrations. DNA duplexes **GC3/15** (A) or **GC12/15** (B) of 0.1, 0.5, 1, 5 or 10 μM was measured in the solution with 1× EvaGreen, 10 mM phosphate (pH 7.4) and 100 mM NaCl.

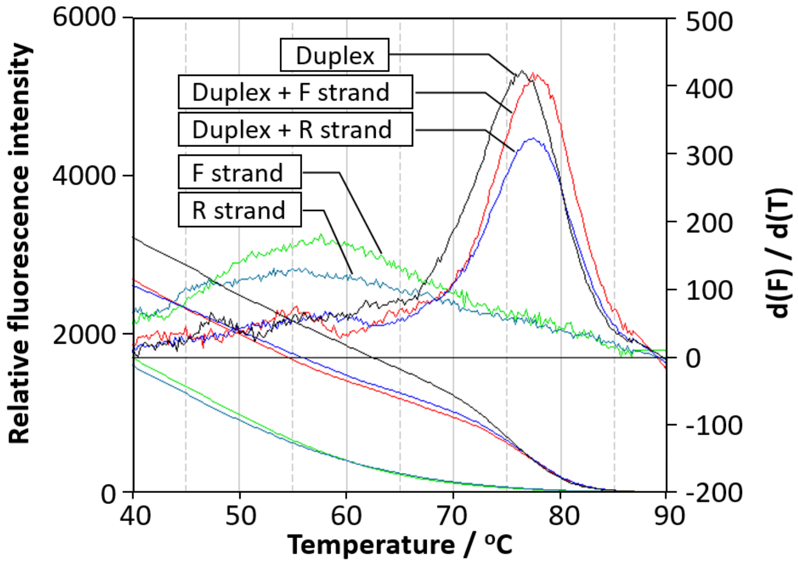

Supplementary Figure S2. Melting curves of **GC14/15** duplex, **GC14/15** duplex with additional F or R strand, and only F strand or R strand. In the assays for **GC14/15** or only F strand or R strand, the concentration of each strand was 1 μM; in the assays for **GC14/15** duplex with additional F or R strand, the concentration of **GC14/15** duplex or additional F or R strand was 1 μM.

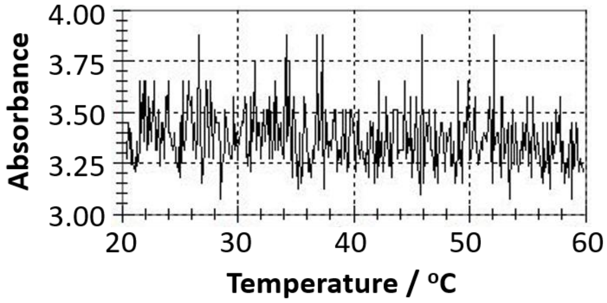

Supplementary Figure S3. Melting measurement of **GC3/15** mixed with 0.2 mM dNTPs by UV melting method.

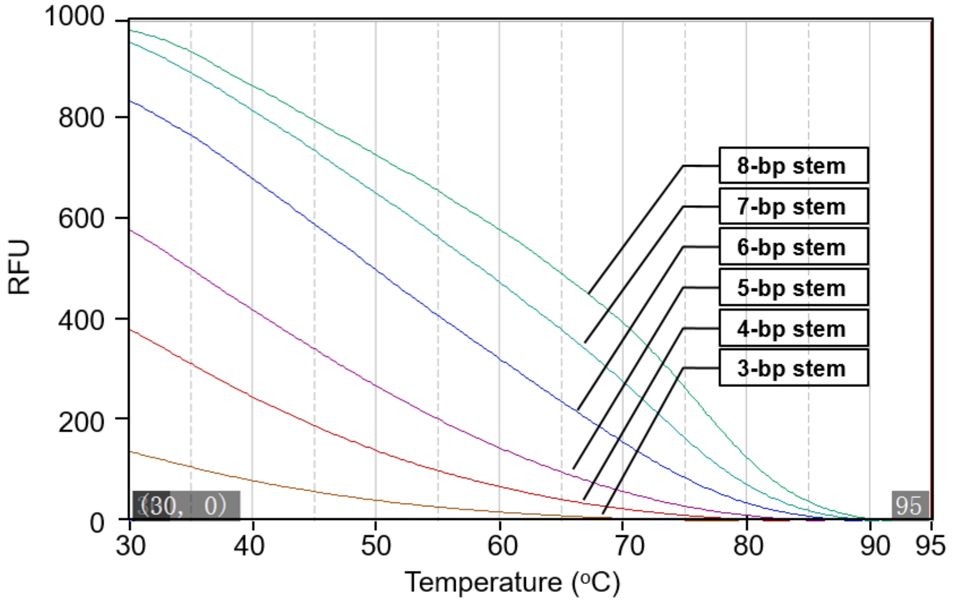

Supplementary Figure S4. Melting curves and their first derivatives for hairpins with 3~8-bp stem obtained by HRM. The left Y-axis displays the detected relative fluorescence units (RFU), and the right Y-axis (-d(RFU)/dT) displays the negative of the first derivative of the melting curve.

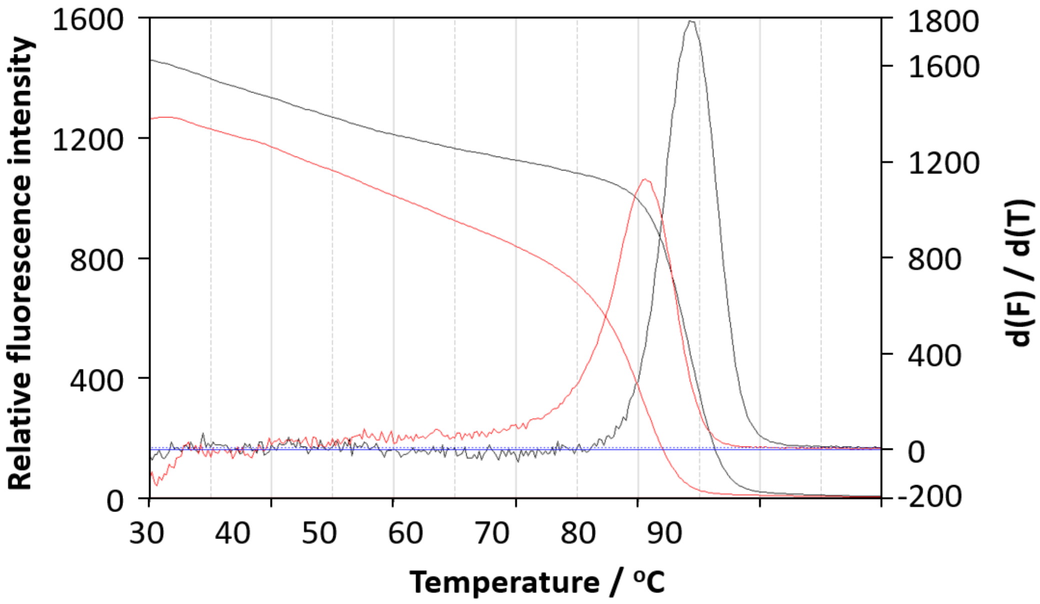

Supplementary Figure S5. Fluorescence melting curves of 21-bp RNA duplex. The RNA duplex (**RR**, sequence in Table 1) was measured at 1 μM (red) and 10 μM (black) in a solution system with 1× EvaGreen, 10 mM phosphate buffer (pH 7.0), 0.5 mM Na_2-EDTA and 100 mM NaCl.

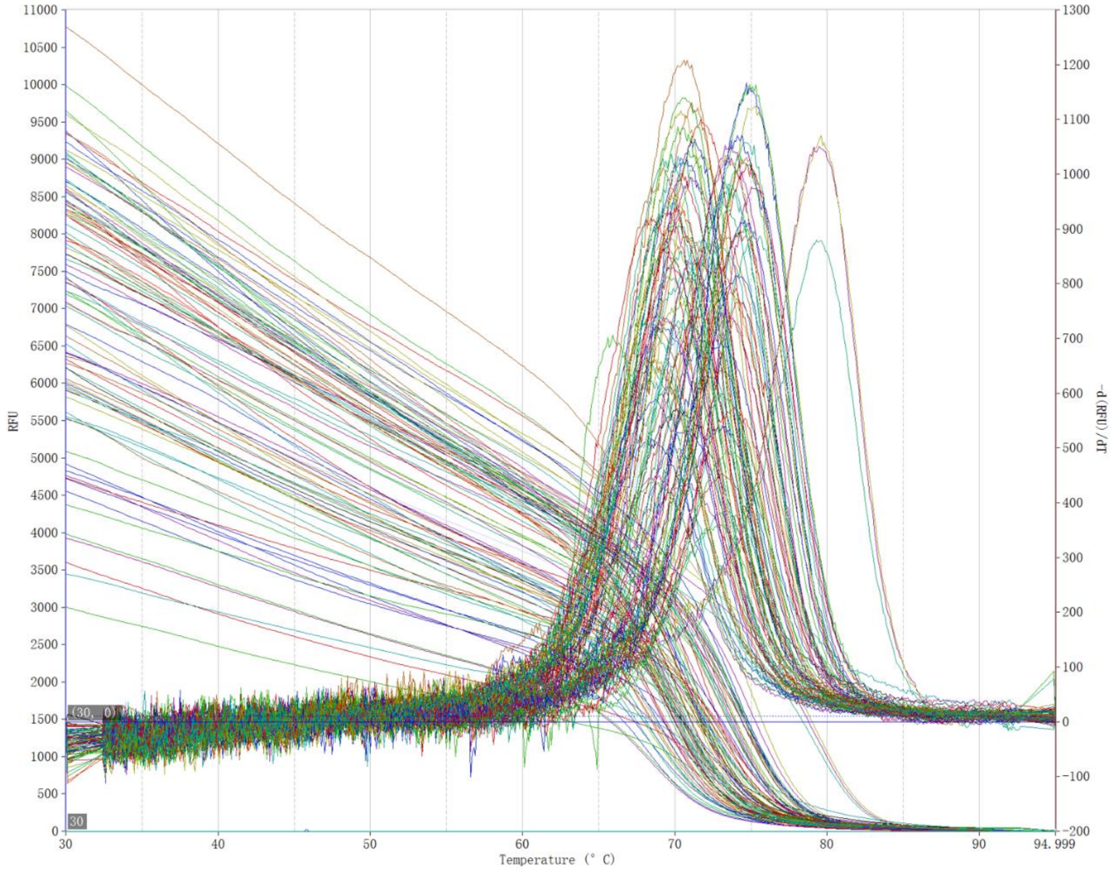

Supplementary Figure S6. Fluorescence melting curves and differential curves of 96 samples on a 96-well plate by HRM. All the data could be obtained in 1 to 2 h. The high throughput can be improved by using 384 well plates.
